# Point-of-care CRP matters: normal CRP levels reduce immediate antibiotic prescribing for acutely ill children in primary care: a cluster randomized controlled trial

**DOI:** 10.1080/02813432.2018.1529900

**Published:** 2018-10-25

**Authors:** Marieke B. Lemiengre, Jan Y. Verbakel, Roos Colman, Kaatje Van Roy, Tine De Burghgraeve, Frank Buntinx, Bert Aertgeerts, Frans De Baets, An De Sutter

**Affiliations:** a Department of Public Health and Primary Care, Ghent University, Ghent, Belgium;; b Nuffield Department of Primary Care Health Sciences, University of Oxford, Oxford, UK;; c Department of Public Health and Primary Care, KU Leuven, Leuven, Belgium;; d Research Institute Caphri, Maastricht University, Maastricht, The Netherlands;; e Department of Pediatric Pulmonology, Infection and Immune Deficiencies, Ghent University Hospital, Ghent, Belgium

**Keywords:** child, acute disease, Anti-Bacterial Agents/therapeutic use, C-reactive protein/analysis, point-of-care-systems, Randomized controlled trial

## Abstract

**Objective:** Antibiotics are prescribed too often in acutely ill children in primary care. We examined whether a Point-of-Care (POC) C-reactive Protein (CRP) test influences the family physicians’ (FP) prescribing rate and adherence to the Evidence Based Medicine (EBM) practice guidelines.

**Design:** Cluster randomized controlled trial.

**Setting:** Primary care, Flanders, Belgium.

**Intervention:** Half of the children with non-severe acute infections (random allocation of practices to perform POC CRP or not) and all children at risk for serious infection were tested with POC CRP.

**Subjects:** Acutely ill children consulting their FP.

**Main outcome measure:** Immediate antibiotic prescribing.

**Results:** 2844 infectious episodes recruited by 133 FPs between 15 February 2013 and 28 February 2014 were analyzed. A mixed logistic regression analysis was performed. Compared to episodes in which CRP was not tested, the mere performing of POC CRP reduced prescribing in case EBM practice guidelines advise to prescribe antibiotics (adjusted odds ratio (aOR) 0.54 (95% Confidence Interval (CI) 0.33–0.90). Normal CRP levels reduced antibiotic prescribing, regardless of whether the advice was to prescribe (aOR 0.24 (95%CI 0.11–0.50) or to withhold (aOR 0.31 (95%CI 0.17–0.57)). Elevated CRP levels did not increase antibiotic prescribing.

**Conclusion:** Normal CRP levels discourage immediate antibiotic prescribing, even when EBM practice guidelines advise differently. Most likely, a normal CRP convinces FPs to withhold antibiotics when guidelines go against their own gut feeling. Future research should focus on whether POC CRP can effectively identify children that benefit from antibiotics more accurately, without increasing the risks of under-prescribing.

**Key points**
What is previously known or believed on this topic
•Antibiotics are prescribed too often for non-severe conditions. Point-of-care (POC) C-reactive Protein (CRP) testing without guidance does not reduce immediate antibiotic prescribing in acutely ill children in primary care.
What this research adds
•FPs clearly consider CRP once available: normal CRP levels discourage immediate antibiotic prescribing, even when EBM practice guidelines advise differently. Most likely, a normal CRP convinces FPs to withhold antibiotics when guidelines go against their own gut feeling.
•Future research should focus on whether POC CRP can effectively identify children that benefit from antibiotics more accurately, without increasing the risks of under-prescribing.

## Introduction

Antibiotics are prescribed too often for acutely ill children in primary care [1]. One possible explanation could be the physicians’ diagnostic uncertainty, as distinguishing between viral and bacterial infections is clinically challenging and denying antibiotics to a child with a possible bacterial infection feels inappropriate [2–6]. To promote appropriate prescribing, evidence-based medicine (EBM) practice guidelines were drawn up [7]. They inform physicians in which specific clinical conditions antibiotics should be prescribed. However, they are not always clear-cut, still leave room for doubt and subjective assessment (e.g. “severe” pain, “less” fluid intake).

C-reactive protein (CRP) has recently been put forward as an objective tool to increase diagnostic certainty. Research has shown that point-of-care (POC) CRP testing improves antibiotic prescribing rates in adults with respiratory tract infections in primary care [8]. In comparable health care settings, no significant reduction has been found in children [9–11]. Aabenhus et al. [8] suggested that when guidance was provided on when to withhold or initiate antibiotic treatment based on specific cut-off values, the effect of POC CRP was more prominent [12–15]. Up to now, such cut-offs in children are lacking.

**Figure 1.**
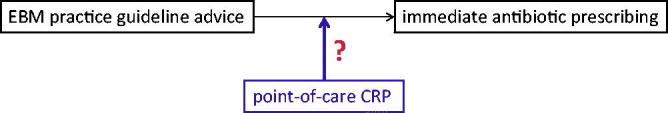
Study rationale.

In the original ERNIE2 trial, we have shown that POC CRP without guidance did not reduce antibiotic prescribing in children with acute non-severe infections in primary care in comparison to usual care [10]. For the present paper, we tested whether family physicians (FP) take CRP into account once measured and to what extent this influences their adherence to the guidelines (Figure 1).

Our hypothesis was that normal CRP levels would support FPs to follow the EBM practice guidelines when the latter advise to withhold antibiotics.

## Methodology

### Study design

We performed an in-depth analysis of the ERNIE2 trial, a cluster randomized controlled trial in children presenting with an acute infection to a FP. Both the study protocol and main results of the ERNIE2 trial have been reported previously [10,16–19].

### Study population

#### Participating family physicians

All FPs in Flanders (Belgium) prepared to consecutively recruit at least five ill children during the inclusion period, were eligible to participate. Details of the recruitment procedure can be found in the published study protocols [16,19].

#### Participating children

Children aged 1 month to 16 years presenting to a participating FP with an acute infection of maximum 5 days were consecutively recruited and received an intervention according to FP’s intervention group. Children at risk for serious infection were identified by means of a clinical prediction rule [17,20] based on four clinical criteria, namely clinician concern (gut feeling ‘something is wrong’), dyspnea, temperature ≥40 °C, and diarrhea in children aged 1 to 2.5 years.

Children referred to secondary care were excluded. Other exclusion criteria were episodes caused by merely traumatic or neurological conditions, intoxication, psychiatric or behavioral problems or an exacerbation of a known chronic condition. Written informed consent was solicited from the child’s accompanying parent or legal guardian.

### Intervention

In the ERNIE2 trial, a POC CRP test was performed in (1) half of the children with acute non-severe infections (scoring ‘no’ on the four criteria of the clinical prediction rule, random allocation of practices to perform POC CRP or not) and (2) all children at risk for serious infection (scoring ‘yes’ (or ‘I don’t know’ in case of clinician concern) on at least one of the criteria of clinical prediction rule). For the present analysis, we compared antibiotic prescribing in children in which CRP was tested to those in which CRP was not tested.

For the POC CRP test (Afinion AS100 Analyzer, Alere, USA), a finger prick was performed and the result was available within 4 minutes [21]. Guidance on the interpretation of CRP-results was not provided, as reliable cut-off values for children in primary care are unknown [11].

### Data collection

FPs registered child characteristics, clinical parameters, preliminary diagnosis and treatment actions (or referral) on a registration form. Parents completed a diary until they deemed their child to be recovered [16].

### End points

The main outcome was the immediate antibiotic prescribing rate. An immediate prescription is meant to be delivered and administered immediately after the consultation.

### Sample size calculation

The original cluster randomized trial was sufficiently powered [10]. Since post hoc power calculations are not useful [22], we did not perform another sample size calculation.

### Statistical analysis

The analyses were performed with SPSS 24 [23]. We performed a mixed effects logistic regression analysis, considering the hierarchical structure of the data (practice level, FP level, patient level).

First, we investigated whether performing POC CRP in itself would influence FP’s adherence to the guidelines. Secondly, we investigated whether this influence was dependent on the CRP level (normal versus elevated).

#### Determining antibiotic prescribing advice provided by the EBM practice guidelines

To determine whether antibiotics were prescribed appropriately, we combined three different elements: (1) preliminary diagnosis (e.g. acute otitis media), (2) clinical signs (e.g. bulging tympanic membrane) and (3) indicators for rational prescribing of antibiotics for a specific diagnosis, following the Belgian national guidelines (e.g. “fever lasting for 3 days or more in children older than 24 months” in case of acute otitis media) (Appendix 1). All diagnoses in which an antibiotic prescription could be recommended by the Belgian Antibiotic Policy Coordination Committee (BAPCOC) were considered, including acute tonsillitis, acute otitis media, acute sinusitis, acute bronchitis, pneumonia, pertussis, impetigo, erysipelas/cellulitis, urinary tract infections and dysentery. The Belgian guidelines [7] are consistent with the European guidelines but adapted to the Belgian bacterial resistance patterns in the choice for an antibiotic class and dosage.

Following this strategy, illness episodes were categorized in three distinct EBM practice guideline advice groups, namely:

- Prescribe antibiotic (e.g. acute otitis media in children younger than 6 months)
- Withhold antibiotic (e.g. acute otitis media in a child older than 24 months without fever)
- No advice, when (1) clinical signs or required indicators were inconclusive, (2) when the FP expressed doubt about the preliminary diagnosis and (3) in case no the preliminary diagnosis was registered.

#### Determining a valuable CRP cut-off

We used the cut-off of 5 mg/L to dichotomize our results to “normal” versus “elevated”.

Besides performing POC CRP and adherence to EBM practice guideline advice, we added 3 covariates to our model to adjust for expected confounding due to the design of the original ERNIE2 trial. *Practice type* was added because we performed stratification at this level. The *result on the clinical prediction rule* was added since (1) FPs knew that this rule identified children at risk for serious infection (as shown before by Van den Bruel et al. [20]) and (2) all these children received a POC CRP test. The *communicative intervention of the original ERNIE2 trial* (applying a brief intervention to elicit parental concern combined with safety net advice (BISNA), performed by half of the FPs) was added considering this intervention increased antibiotic prescribing [10].

After performing the partially adjusted analysis, we adjusted our analyses further for other covariates that could have influenced antibiotic prescribing too.

At *practice level*, we considered geographical region (urban/rural).

At *FP level*, we investigated the role of personal characteristics (FP’s gender, age, years of experience), annual antibiotic prescription rate as provided by the Quality assurance initiative of the Belgian social security authority (RIZIV-INAMI) and an indicator of the risk-avoiding attitude. We used the individual annual antibiotic prescription rate during 2011 (proportion of patients allocated to a FP who got an antibiotic prescription during that year, children and adults, most recent Belgian data available) as a proxy for baseline antibiotic prescribing. National prescribing data for children only were not available. We categorized FPs as high or low prescribers (with the national mean as threshold). As prescription data from early career FPs and residents were not yet available, we considered them as a separate group. All FPs completed a validated questionnaire (5 questions) measuring their risk-avoiding behavior (also called ‘defensive attitude’). The higher the sum score of this questionnaire (range 5 to 25), the more the FP will prefer the certain to the uncertain [24].

At *child level*, we considered age (infant, preschool child, child/adolescent) and fever (no fever, elevation, high fever (39 °C or more)). We also considered the perceived parental expectation regarding antibiotics, which was registered by the FPs at the end of the consultation by answering “yes/no” to the question: “Do you think this parent expects antibiotic treatment?”. The option “I don’t know” or missing values were categorized as “unknown”.

First, we explored which of these covariates at the univariate level influenced immediate antibiotic prescribing when added to the partially adjusted analysis. Secondly, covariates with p-values lower than 0.1 were included in the fully adjusted analysis, in addition to the covariates for which limited imbalances between the intervention groups were found: the child’s age and temperature.

## Results

### Participant flow, recruitment and numbers analyzed

**Figure 2.**
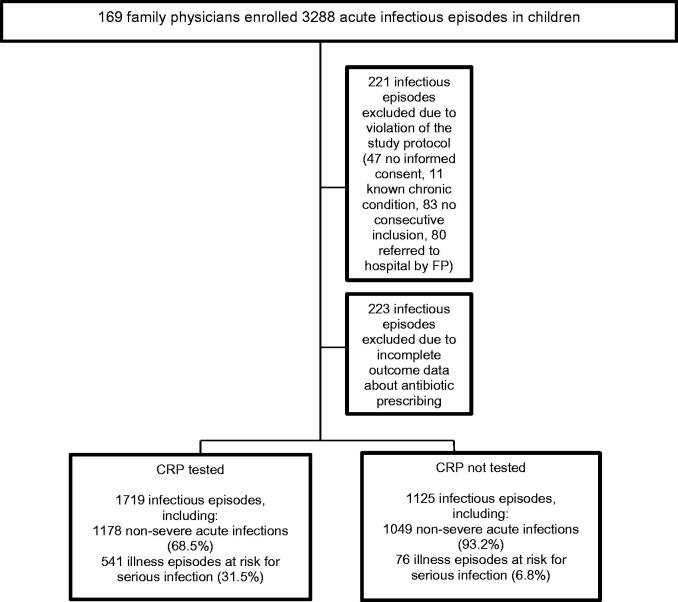
Flow chart representing the number of acute infectious episodes included in the study.

169 FPs started recruitment. Initially, 3288 acute infectious episodes were included between 15 February 2013 and 28 February 2014. After the application of the exclusion criteria (Figure 2), 2844 acute infectious episodes registered by 133 FPs (79 practices) were analyzed. 34 physicians were excluded because they included less than 5 children. Their baseline characteristics were comparable to those of included FPs (Appendix 2). 223 episodes were discarded because of missing data on antibiotic prescribing. These children were of similar age but had less often fever (*p* < .001) and represented more cases in which no guideline advice could be provided (*p* < .001) in comparison to children of whom outcome data on antibiotic prescribing was available (Appendix 3). Illness episodes in which performing POC CRP failed (no registered CRP value) were added to the 'CRP not tested' group.

### Baseline characteristics

#### Family physicians

55 FPs (41.4%) were men. The mean age was 39.9 (standard deviation (SD) 10.7) and 17.3% were in postgraduate training. Forty percent were practicing in an urban region. The mean risk-avoiding behavior was 17.3 (SD 3.0 on a scale of 5 to 25), and the mean annual antibiotic prescription rate was 42.1% (SD 9.5). The median number of included infectious episodes per FP was 14 (interquartile range (IQR) 8 to 23.5, range 3 to 342).

#### Children

Fifty-two percent of infectious episodes concerned boys. The mean age was 4.6 years (SD 4.2, IQR 1.4 to 6.8; 34.0% infant, 37.9% preschool child, 28.2% child/teenager). 37.5% suffered from high fever (39 °C or more). The top 3 preliminary diagnoses were: upper respiratory tract infection (31.0%), acute otitis media (15.3%) and other viral disease (11.1%) (Appendix 3). 617 children (21.6%) were at risk for serious infection (according to the clinical prediction rule). As expected, there were imbalances in age, temperature and the presence of an appropriate indication for antibiotics between children in which CRP was tested or not tested (Appendix 4). This could be largely explained by the fact that all children at risk for serious infection according to the clinical prediction rule received a POC CRP test and were therefore assigned to the 'CRP tested' group. Children at risk for serious infection were generally younger, had more frequently high fever and suffered more from bronchitis and pneumonia, which was consequently reflected in the higher number of episodes in which antibiotics were advised by the EBM practice guidelines (Appendix 5). In children with acute non-severe infections, some small imbalances in age and fever between the study arms remained, but there was no longer a difference in the number of illness episodes in which EBM practice guidelines advised to prescribe antibiotics (Appendix 5).

For 1287 episodes (45.3%), the parents returned the diary. There were no differences in baseline characteristics of children whose parents did or did not return the diary, except a minor difference in the child’s temperature: the number of children with high fever was slightly larger when the diary was returned (39.2% versus 36.1%).

### Outcomes and estimates

In 1719 illness episodes (59.6%), CRP levels were measured. CRP levels were normal in 693 illness episodes (40.3%). Elevated CRP levels varied from 5 to 201 mg/L (mean 30.3 (SD 32.3), IQR 9-38). CRP was more frequently elevated when the EBM practice guidelines advised to prescribe antibiotics in comparison to those episodes in which they advised to withhold antibiotics (77.6% versus 52.9%, *p* < .001).

In 561 episodes (19.7%), FPs prescribed antibiotics immediately. In 2410 illness episodes (84.7%), the collected data provided enough information to determine the antibiotic prescribing advice based on the EBM practice guidelines. In 2013 illness episodes (83.5%), FPs followed the antibiotic prescribing advice. In 175 illness episodes (7.3%), overprescribing was detected. In 222 illness episodes (9.2%), under-prescribing was detected: FPs withheld antibiotics when the EBM practice guidelines advised to prescribe immediately (Table 1).

**Table 1.** Observed immediate antibiotic prescribing rates differ depending on (1) performance and/or result of POC CRP test and (2) EBM practice guideline advice.

	POC CRP test			
	CRP not tested	Normal CRP level (<5 mg/L)	Elevated CRP level (≥5 mg/L)	Total
Advice				
Prescribe antibiotic	88/149 (59.1%)	24/74 (32.3%)	146/257 (56.8%)	258/480 (53.6%)
Withhold antibiotic	71/824 (8.6%)	17/521 (3.2%)	87/585 (14.9%)	175/1930 (9.1%)
No advice	44/152 (28.9%)	9/98 (9.2%)	75/184 (40.7%)	128/434 (29.4%)
*Total*	203/1125 (18.0%)	50/693 (7.2%)	308/1026 (30.0%)	561/2844 (19.7%)

The intracluster correlation coefficient (ICC) was low (8.9% at practice level and 1.9% at FP level).

The partially and fully adjusted mixed logistic regression analysis showed that performing POC CRP in itself influenced adherence to the guideline: when the EBM practice guidelines advise to prescribe antibiotics (A), mere performing of POC CRP (regardless of the result) reduced prescribing (partially adjusted analysis: odds ratio (OR) 0.52 (95% Confidence Interval (CI) 0.32–0.84); fully adjusted analysis: adjusted odds ratio (aOR) 0.54 (95% CI 0.33–0.90)). This was not the case when EBM practice guidelines advise to withhold antibiotics (B) (partially adjusted analysis: OR 0.86 (95% CI 0.58–1.27); fully adjusted analysis: aOR 0.81 (95% CI 0.54–1.20)).

**Figure 3.**
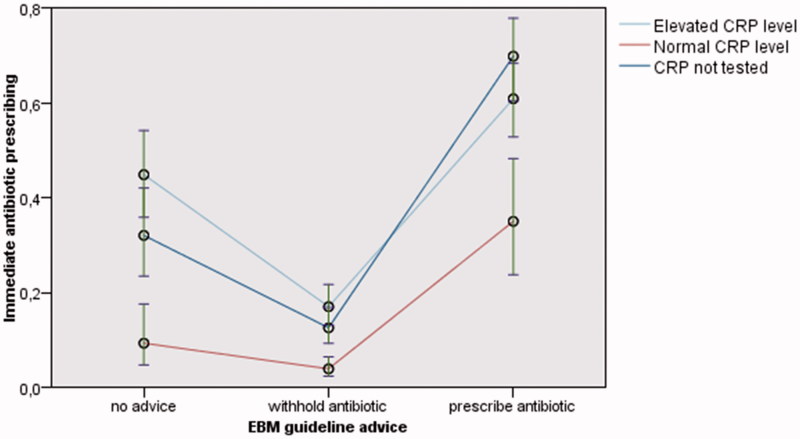
Estimated marginal means of the immediate antibiotic prescribing rate (with 95% confidence interval) according to EBM guideline advice and POC CRP testing (partially adjusted analysis). EBM: Evidence Based Medicine; POC CRP: point-of-care C-reactive protein.

Focusing on the result of the POC CRP test (normal or elevated), the partially adjusted and fully adjusted mixed logistic regression analysis showed that normal CRP levels further reduced antibiotic prescribing whatever EBM practice guidelines advise (fully adjusted analysis: “prescribe antibiotics (A)”: aOR 0.24 (95% CI 0.11–0.50); “withhold antibiotics (B)”: aOR 0.31 (95% CI 0.17–0.57); “no advice (C)”: aOR 0.26 (95% CI 0.11–0.64)). Elevated CRP levels did not significantly increase prescribing, except when no advice (C) could be given because the preliminary diagnosis and/or clinical indicators were lacking or unsure (borderline significance) (fully adjusted analysis: “no advice (C)”: aOR 1.82 (95% CI 1.01–3.27)) (Figure 3, Table 2, Appendix 6).

**Table 2.** Influence of POC CRP (normal or elevated versus not measured) on immediate antibiotic prescribing according to EBM guideline advice.

	Partially adjusted analysis^b^		Fully adjusted analysis^c^	
	OR	95%CI	OR	95%CI
Advice: “Prescribe antibiotic (A)” (480 illness episodes)				
Normal CRP level	*0.23*	*0.12–0.45*	*0.24*	*0.11–0.50*
Elevated CRP level	0.67	0.41–1.11	0.69	0.40–1.17
CRP not tested^a^				
Advice: “Withhold antibiotic (B)” (1930 illness episodes)				
Normal CRP level	*0.28*	*0.16–0.51*	*0.31*	*0.17–0.57*
Elevated CRP level	1.43	0.95–2.15	1.23	0.80–1.89
CRP not tested^a^				
No advice (C) (434 illness episodes)				
Normal CRP level	*0.22*	*0.10–0.50*	*0.26*	*0.11–0.64*
Elevated CRP level	1.73	1.01–2.96	*1.82*	*1.01–3.27*
CRP not tested^a^				

^a^Reference category.

^b^2844 illness episodes analyzed. Adjusted for performance of the BISNA-intervention, risk for serious infection estimated by a clinical prediction rule, practice type.

^c^2804 illness episodes analyzed (40 episodes (1.4%) excluded because missing information about temperature). Adjusted for applying the BISNA-intervention, risk for serious infection estimated by a clinical prediction rule, practice type, region, mean annual antibiotic prescription rate, perceived parental expectation regarding antibiotics, child’s age, temperature.

Sensitivity analyses for children with acute non-severe infections versus children at risk for serious infections (according to the clinical prediction rule), followed the same trend (Appendix 7 and 8).

#### Harms

All children recovered. Based on data from the diaries, on average, children were better in 4 days (SD 3.9, IQR 2.0–5.0). 7 children (0.3%) were hospitalized for a serious infection. All these children were identified by the clinical prediction rule as at risk for serious infection and no hospitalization was due to unjustly withholding antibiotics.

## Discussion

### Main findings

Although the use of POC CRP without guidance did not reduce immediate antibiotic prescribing [10], FPs clearly consider CRP once available: independent of what guidelines advise, normal CRP levels are a strong argument for FPs to withhold antibiotics. POC CRP discourages antibiotic prescribing even when EBM practice guidelines advise the opposite. This finding is extraordinary, since there is at this moment still no evidence that CRP can reliably discriminate viral from bacterial infections in acute infections in children [25–27].

### Strengths and limitations

Our databank consists of a large unique collection of illness episodes representative for infections commonly seen in children by FPs. The registered clinical data provided sufficient information to formulate a guideline based antibiotic prescribing advice in 85% of illness episodes, which makes our results solid.

The observed antibiotic prescribing rate was low (31.9%, of which 19.7% immediate prescriptions). Based on data from a Belgian continuous and integrated computerized morbidity registration network (INTEGO) [28], we expected a prescribing rate of 40%. FPs may have inclined to avoid prescribing antibiotics during the trial because they were eager to perform well [29]. The low prescribing rate in our study cannot be explained by the selection of FPs since their mean annual antibiotic prescription rate was comparable to the national mean. Moreover, 31.2% of the immediate prescriptions could still be considered as inappropriate which highlights the room for improvement.

The Belgian BAPCOC guidelines [7] formed the basis to assess the EBM practice guideline advice, since these are the ones that should be followed by the participating FPs. These guidelines are consistent with the European guidelines but adapted to the Belgian bacterial resistance patterns. In comparison to the US guidelines, these recommendations are more restrictive in indications for antibiotic treatment for acute otitis media (considering age, clinical signs, risk factors for complications or severe illness) and pharyngitis (no common practice to test for streptococcus A infection) [30,31].

The assessment of the EBM practice guideline advice was done following strict predetermined rules but was inevitably dependent on the quality of the registered information. The preliminary diagnoses registered by the FP, were classified independently by two investigators to avoid imprecision bias in coding. Moreover, our strategy dealt effectively with the risk of ‘diagnostic labeling’ (when FPs register a specific preliminary diagnosis to justify their antibiotic prescription) since a diagnostic label was not sufficient to decide whether there was an appropriate indication for antibiotics. For the analyses we separated illness episodes in which there was insufficient information to reconstruct any advice.

In 10.9% (310/2844) of the infectious episodes, FPs thought that parents expected antibiotics. Although we did not find a significant difference concerning this covariate between infectious episodes with known and unknown CRP levels (10.9% (188/1719) versus 10.8% (122/1125), *p* = .71), the analyses were corrected for this covariate because it is known that the perceived expectation strongly influences antibiotic prescribing [32–35]. We aimed to estimate the effect of knowing CRP levels on antibiotic prescribing, independent of the influence of the perceived parental expectation for antibiotics.

We used the threshold of 5 mg/L to dichotomize the results of the POC CRP test (normal versus elevated). Previously, the ERNIE2 trial showed that when adding a POC CRP test to a clinical prediction rule, this threshold was useful to further exclude serious illness in primary care [17,18]. For other proposed thresholds, varying from 10 to 50 mg/L, there is no good evidence in primary care that these are useful to exclude serious illness, nor to differentiate between viral and bacterial disease [11]. Our trial did not have enough power to examine subgroups based on different thresholds.

Because of the original trial design in which all children at risk for serious infection had a POC CRP test, the group of children with known CRP levels was somewhat different from the group of children in which CRP was not tested. Still, adjusting for these imbalances and performing sensitivity analyses for children at low versus higher risk for serious infection did not change our overall result.

Systematic POC CRP testing was performed in half of the children according to the protocol of the ERNIE2-trial in order to answer its divergent research questions [16,19]. This practice does not replicate clinical work in primary care. In case of no uncertainty, physicians have no need to use additional testing and should be able to reassure parents without antibiotic prescription. Furthermore, introducing systematic POC CRP testing could lead to high cost for the health insurance: (1) the device as the test kits are expensive, and (2) when the CRP level is not congruent with the FP’s clinical appraisal, this can further lead to unnecessary additional testing and referrals.

Our results are generalizable to similar children in other developed countries. However, results could be different in countries with lower health care accessibility.

### Comparison with existing literature

We did not find any trials investigating the effect of using POC CRP on the adherence to the antibiotic prescribing guidelines. Other trials investigating the adherence to guidelines, focused on the relation between a prescription and a certain diagnosis [36–41], but not on the effect of the introduction of new technology on guideline adherence.

### Implications for research and/or practice

FPs tend to withhold antibiotics for acute infections with normal CRP levels, even when EBM practice guidelines advice to prescribe. In our trial, we have no arguments to state that this finding is caused by guideline unawareness, since the adherence of participating FPs to these guidelines was remarkably high (83.5%). The finding that POC CRP discourages antibiotic prescribing when EBM practice guidelines advise differently, gives a cause for concern as it may result in under-prescribing and could be dangerous, since a normal CRP during the doctors’ visit is no guarantee for an uncomplicated illness course. However, in our trial, all children recovered, and no hospitalization for serious infection could be attributed to under-prescribing. EBM guidelines are vague at some points. Probably, normal CRP levels tipped the scale to withhold antibiotics in illness episodes in which the EBM practice guideline advice leaves too much room for doubt. We hypothesize that a normal CRP offered FPs a convincing argument to withhold antibiotic in those illness episodes where advice to prescribe antibiotics went against their own gut feeling.

Evidence based guidelines should form the basis for the clinical decision making. However, in this in-depth analysis, we found that FPs seem to be receptive for a tangible argument such as POC CRP. Up to now, POC CRP was never explored to narrow the safety margins of the guidelines or make the instructions more clear-cut. Future research should focus on whether POC CRP can effectively help to more accurately identify children who might benefit from antibiotics without increasing the risks of under-prescribing.

## Trial registration

ClinicalTrials.gov Identifier: NCT02024282

## Appendix Appendix 1: Rational indications for antibiotics according to the Belgian guidelines (BAPCOC)

**Table:**

Preliminary diagnosis	Required clinical symptom	Required criteria for rational prescribing
Acute tonsillitis	Pus on tonsils	Appearing seriously ill or less eating/drinking
Acute otitis media	Clinical signs of acute otitis media	1. < 6 months OR 2. 6–24 months and appearing seriously ill, fever lasting at least 2 days or bilateral AOM OR 3. >24 months and persistent fever lasting at least 3 days or appearing seriously ill OR 4. Persistent otorrhea
Acute sinusitis	No clinical symptom required	1. Appearing seriously ill OR 2. Fever ≥39 °C OR 3. Red swollen face or eyelid (referral)
Pneumonia	No clinical symptom required	No criteria required
Acute bronchitis	No clinical symptom required	1. < 3 months (referral) OR 2. < 24 months and insufficient fluid intake or breathing rate >70/’ (referral) OR 3. Suspected for serious infection (insufficient fluid intake and vomiting, breathing rate >50/’, oxygen saturation ≤92%, sleepy, waking up hard, irritable, drowsy, reduced consciousness, moaning, nasal flaring or chest wall retracting) (referral)
Pertussis	No clinical symptom required	No criteria required
Impetigo	No clinical symptom required	Fever ≥38.5 °C, adenopathy or failure local therapy
Erysipelas	No clinical symptom required	No criteria required
Urinary tract infection (cystitis or pyelonephritis)	No clinical symptom required	No criteria required
Gastrointestinal infection with diarrhea	Diarrhea	Fever ≥38.5 °C, bloody diarrhea or appearing seriously ill

BAPCOC: Belgian Antibiotic Policy Coordination Committee.

## Appendix Appendix 2: Comparison of characteristics of included and excluded FP (after randomization)

**Table:**

Characteristic	Included FP (133)	Excluded FP (34)	*p*
	*n* (%) / mean (SD)	*n* (%) / mean (SD)	
Gender (man)	55 (41.4%)	14 (41.2%)	.99^1^
Age	39.9 (10.7)	38.7 (13.2)	.57^2^
Region (urban)	53 (39.8%)	8 (25.0%)	.18^1^
Graduated (resident)	23 (17.3%)	7 (21.2%)	.60^1^
Years of experience	13.3 (10.8)	12.3 (13.0)	.66^2^
Practice type (exclusive residents)			.14^1^
• Solo	19 (14.3%)	8 (23.5%)	
• Duo	45 (33.8%)	6 (17.6%)	
• Group	69 (51.9%)	20 (58.8%)	

^1^Chi-square test

^2^Student t test

FP: family physician; SD: standard deviation.

## Appendix Appendix 3: Comparison of characteristics of infectious episodes with and without outcome data

**Table:**

Characteristic	Included infectious episode (2844)	Excluded infectious episodes (223)	*p*
	*n* (%)	*n* (%)	
Age^$^			
Infant	966 (34.0%)	69 (31.5%)	.16
Preschool child	1077 (37.9%)	75 (34.2%)	
Child	801 (28.2%)	75 (34.2%)	
Temperature^$^			
No fever	626 (22.3%)	76 (36.5%)	<.001
Elevation	1127 (40.3%)	76 (36.5%)	
High fever (39° or more)	1051 (37.5%)	56 (26.9%)	
CRP			
Tested	1719 (60.4%)	129 (57.8%)	.45
Not tested	1125 (39.6%)	94 (42.2%)	
EBM guideline advice			
Prescribe antibiotic (A)	480 (16.9%)	8 (3.6%)	<.001
Withhold antibiotic (B)	1930 (67.9%)	151 (67.7%)	
No advice (C)	434 (15.3%)	64 (28.7%)	

$ 4 cases no information about age (4 excluded infectious episodes), 55 cases no information about temperature (40 included infectious episodes, 15 excluded infectious episodes).

## Appendix Appendix 4: Comparison of characteristics of infectious episodes with tested CRP versus those in which CRP was not tested

**Table:**

Characteristic	CRP tested (1719)	CRP not tested (1125)	*p*
	*n* (%)	(%)	
Age			
Infant	581 (33.8%)	385 (34.2%)	.002
Preschool child	615 (35.8%)	462 (41.1%)	
Child	523 (30.4%)	278 (24.7%)	
Temperature^$^			
No fever	400 (23.3%)	226 (20.1%)	.001
Elevation	634 (36.9%)	493 (43.8%)	
High fever (39° or more)	664 (38.6%)	387 (34.4%)	
EBM guideline advice			
Prescribe antibiotic (A)	331 (19.3%)	149 (13.2%)	<.001
Withhold antibiotic (B)	1106 (64.3%)	824 (73.2%)	
No advice (C)	282 (16.4%)	152 (13.5%)	
Top 10 preliminary diagnoses			
Upper respiratory tract infection, acute	482 (28.0%)	401 (35.6%)	NA
Acute otitis media	276 (16.1%)	158 (14.0%)	
Viral disease, other (NOS)	197 (11.5%)	119 (10.6%)	
Gastroenteritis, presumed infection	160 (9.3%)	93 (8.3%)	
Bronchitis (no bronchiolitis)	183 (10.6%)	67 (6.0%)	
Tonsillitis	119 (6.9%)	96 (8.5%)	
Influenza	111 (6.5%)	56 (5.0%)	
Pneumonia	67 (3.9%)	15 (1.3%)	
Urinary tract infection	42 (2.4%)	15 (1.3%)	
Bronchiolitis	23 (1.3%)	9 (0.8%)	
No preliminary diagnosis registered	103 (6.0%)	63 (5.6%)	
Diary return	777 (45.2%)	510 (45.3%)	.945

$ 40 infectious episodes were excluded because of missing information about temperature.

## Appendix Appendix 5: Comparison of characteristics of infectious episodes with tested CRP versus those in which CRP was not tested, according to the risk for serious infection

**Table:**

	Non-severe acute infections		Illness episodes at risk for serious infection	
Characteristic	CRP tested	CRP not tested	CRP tested	CRP not tested
	(1178)	(1049)	(541)	(76)
Age				
Infant	330 (28.0%)	351 (33.5%)	251 (46.4%)	34 (44.7%)
Preschool child	404 (34.3%)	431 (41.1%)	211 (39.0%)	31 (40.8%)
Child	444 (37.7%)	267 (25.5%)	79 (14.6%)	11 (14.5%)
Temperature^$^				
No fever	349 (30.1%)	219 (21.2%)	51 (9.5%)	7 (9.5%)
Elevation	495 (42.7%)	473 (45.8%)	139 (25.8%)	20 (27.0%)
High fever (39° or more)	315 (27.2%)	340 (32.9%)	349 (64.7%)	47 (63.5%)
				
EBM guideline advice				
Prescribe antibiotic (A)	161 (13.7%)	130 (12.4%)	170 (31.4%)	19 (25.0%)
Withhold antibiotic (B)	850 (72.2%)	788 (75.1%)	256 (47.3%)	36 (47.4%)
No advice (C)	167 (14.2%)	131 (12.5%)	115 (21.3%)	21 (27.6%)
Top 10 preliminary diagnoses				
Upper respiratory tract infection, acute	384 (32.6%)	383 (36.5%)	98 (18.1%)	18 (23.7%)
Acute otitis media	193 (16.4%)	152 (14.5%)	83 (15.3%)	6 (7.9%)
Viral disease, other (NOS)	147 (12.5%)	115 (11.0%)	50 (9.2%)	4 (5.3%)
Gastroenteritis, presumed infection	101 (8.6%)	80 (7.6%)	59 (10.9%)	13 (17.1%)
Bronchitis (no bronchiolitis)	89 (7.6%)	60 (5.7%)	94 (17.4%)	7 (9.3%)
Tonsillitis	84 (7.1%)	88 (8.4%)	35 (6.5%)	8 (10.5%)
Influenza	84 (7.1%)	50 (4.8%)	27 (5.0%)	6 (7.9%)
Pneumonia	18 (1.5%)	9 (0.9%)	49 (9.1%)	6 (7.9%)
Urinary tract infection	22 (1.9%)	11 (1.0%)	20 (3.7%)	4 (5.3%)
Bronchiolitis	5 (0.4%)	8 (0.8%)	18 (3.3%)	1 (1.3%)
No preliminary diagnosis registered	50 (4.2%)	54 (5.3%)	53 (9.8%)	9 (11.8%)

$ 40 infectious episodes were excluded because of missing information about temperature (36 in children with non-serious acute infections, 4 in children at risk for serious infection).

## Appendix Appendix 6: Influence of POC CRP (normal or elevated versus not tested) on immediate antibiotic prescribing depending on EBM guideline advice (final adjusted analysis, complete model)

**Table:**

	Fully adjusted analysis^$^	
	OR	95%CI
Advice: “Prescribe antibiotic (A)” (480 illness episodes)		
Normal CRP level	*0.24*	*0.11–0.50*
Elevated CRP level	0.69	0.40–1.17
CRP not tested*		
Advice: “Withhold antibiotic (B)” (1930 illness episodes)		
Normal CRP level	*0.31*	*0.17–0.57*
Elevated CRP level	1.23	0.80–1.89
CRP not tested*		
No advice (C) (434 illness episodes)		
Normal CRP level	*0.26*	*0.11–0.64*
Elevated CRP level	*1.82*	*1.01–3.27*
CRP not tested*		
Practice type		
Group	1.22	0.76–1.97
Duo	0.88	0.54–1.42
Solo*		
Clinical prediction rule		
Children at risk for serious infection	2.08	1.54–2.81
Children with non-severe acute infections*		
BISNA		
BISNA performed	1.29	0.91–1.83
BISNA not performed*		
Region		
Urban	0.95	0.66–1.37
Rural*		
Mean annual antibiotic prescription rate		
Resident/Early career FP	1.78	1.12–2.82
High prescriber	2.21	1.48–3.31
Low prescriber*		
Perceived parental expectation regarding antibiotics		
Unknown	2.70	1.95–3.73
Present	12.10	8.60–17.00
Absent*		
Child’s age		
Infant	0.72	0.51–1.01
Preschool	0.99	0.72–1.36
Child/Adolescent*		
Temperature		
High fever	2.09	1.37–3.19
Elevation	1.58	1.05–2.37
No fever*		

*Reference category.

$ 40 infectious episodes were excluded because of missing information about temperature.

## Appendix Appendix 7: Influence of POC CRP (normal or elevated versus not tested) on immediate antibiotic prescribing depending on guideline advice in children with non-severe acute infections

**Table:**

	Partially adjusted analysis^$1^		Fully adjusted analysis^$2^	
	OR	95%CI	OR	95%CI
Advice: “Prescribe antibiotic (A)” (291 illness episodes)				
Normal CRP level	0.28	0.12–0.64	0.27	0.11–0.66
Elevated CRP level	0.55	0.29–1.01	0.65	0.34–1.25
CRP not tested*				
Advice: “Withhold antibiotic (B)” (1638 illness episodes)				
Normal CRP level	0.26	0.13–0.54	0.28	0.13–0.60
Elevated CRP level	1.58	0.95–2.63	1.44	0.86–2.40
CRP not tested*				
No advice (C) (298 illness episodes)				
Normal CRP level	0.11	0.03–0.40	0.14	0.04–0.56
Elevated CRP level	1.28	0.64–2.57	1.38	0.65–2.92
CRP not tested*				

*Reference category

^$1^2227 illness episodes analyzed. Adjusted for performance of the BISNA-intervention, risk for serious infection estimated by a clinical prediction rule, practice type.

^$2^2191 illness episodes analyzed (36 episodes (1.6%) excluded because missing information about temperature). Adjusted for performance of the BISNA-intervention, practice type, region, mean annual antibiotic prescription rate, perceived parental expectation regarding antibiotics, child’s age, temperature.

## Appendix Appendix 8: Influence of POC CRP (normal or elevated versus not tested) on immediate antibiotic prescribing depending on guideline advice in children at risk for serious infection

**Table:**

	Partially adjusted analysis^$1^		Fully adjusted analysis^$2^	
	OR	95%CI	OR	95%CI
Advice: “Prescribe antibiotic (A)” (189 illness episodes)				
Normal CRP level	0.25	0.07–0.93	0.46	0.11–1.92
Elevated CRP level	1.30	0.45–3.79	1.74	0.54–5.58
CRP not tested*				
Advice: “Withhold antibiotic (B)” (292 illness episodes)				
Normal CRP level	0.25	0.07–0.86	0.32	0.09–1.14
Elevated CRP level	1.02	0.40–2.64	0.96	0.35–2.65
CRP not tested*				
No advice (C) (136 illness episodes)				
Normal CRP level	0.50	0.13–1.91	0.45	0.10–1.98
Elevated CRP level	2.69	0.93–7.81	2.29	0.69–7.65
CRP not tested*				

*Reference category

$1 617 illness episodes analyzed. Adjusted for performance of the BISNA-intervention, risk for serious infection estimated by a clinical prediction rule, practice type.

$2 613 illness episodes analyzed (4 episodes (0.6%) excluded because missing information about temperature). Adjusted for performance of the BISNA-intervention, practice type, region, mean annual antibiotic prescription rate, perceived parental expectation regarding antibiotics, child’s age, temperature.
